# Exploration and validation of radiomics signature as an independent prognostic biomarker in stage III-IVb nasopharyngeal carcinoma

**DOI:** 10.18632/oncotarget.20423

**Published:** 2017-08-24

**Authors:** Fu-Sheng Ouyang, Bao-Liang Guo, Bin Zhang, Yu-Hao Dong, Lu Zhang, Xiao-Kai Mo, Wen-Hui Huang, Shui-Xing Zhang, Qiu-Gen Hu

**Affiliations:** ^1^ Department of Radiology, The First People’s Hospital of Shunde, Foshan, Guangdong, P.R. China; ^2^ Medical Imaging Center, First Affiliated Hospital of Jinan University, Guangzhou, Guangdong, P.R. China; ^3^ Institute of Molecular and Functional Imaging, Jinan University, Guangzhou, Guangdong, P.R. China; ^4^ Department of Radiology, Guangdong General Hospital/Guangdong Academy of Medical Sciences, Guangzhou, Guangdong, P.R. China

**Keywords:** nasopharyngeal carcinoma, radiomics signature, prognostic, biomarker

## Abstract

There is no consensus on specific prognostic biomarkers potentially improving survival of nasopharyngeal carcinoma (NPC), especially in advanced-stage disease. The prognostic value of MRI-based radiomics signature is unclear. A total of 970 quantitative features were extracted from the tumor of 100 untreated NPC patients (stage III-IVb) (discovery set: n = 70, validation set: n = 30). We then applied least absolute shrinkage and selection operator (lasso) regression to select features that were most associated with progression-free survival (PFS). Candidate prognostic biomarkers included age, gender, overall stage, hemoglobin, platelet counts and radiomics signature. We developed model 1 (without radiomics signature) and model 2 (with radiomics signature) in the discovery set and then tested in the validation set. Multivariable Cox regression analysis was used to yield hazard ratio (HR) of each potential biomarker. We found the radiomics signature stratified patients in the discovery set into a low or high risk group for PFS (HR = 5.14, *p* < 0.001) and was successfully validated for patients in the validation set (HR = 7.28, *p* = 0.015). However, the other risk factors showed no significantly prognostic value (all p-values for HR, > 0.05). Accordingly, pretreatment MRI-based radiomics signature is a non-invasive and cost-effective prognostic biomarker in advanced NPC patients, which would improve decision-support in cancer care.

## INTRODUCTION

Nasopharyngeal carcinoma (NPC) is a unique cancer with specific patterns of racial and geographical distribution [1]. It is especially prevalent in southern China. The annual incidence rate of NPC reaches approximately 50 per 100,000 in prevalent regions and this value is 50-fold higher than that in western countries [2]. Although NPC is radio-sensitive, about 70-80% patients have locoregionally advanced disease at diagnosis and 20-30% of NPC patients experience treatment failure [3, 4]. The main causes of treatment failure are locoregional recurrences and distant metastasis [5].

While concurrent chemoradiotherapy with or without adjuvant chemotherapy has led to gains in overall survival of advanced NPC patients, there is also wider recognition that the outcome of these patients are clinically heterogeneous [6]. When they are stratified by clinical stage, differences in long-term survival are evident within the individual stages. If poor survival can be identified pre-treatment, then this can attribute to determine whether more aggressive treatments should be administered, for example by increasing cycles, or by using of adjuvant chemotherapy. To date, plasma cell-free EBV DNA titre remains the only biomarker with clinical utility in NPC [7, 8]. There is, thus, a critical need for additional biomarkers for prognostication and precise treatment stratification in advanced NPC patients.

Recent advances in imaging analysis have allowed noninvasive, three dimensional and quantitative characterization of tumor with a great potential for therapy guidance by providing a comprehensive view of the whole tumor, accounting for intratumoral heterogeneity, and unrestricted repeatability during the course of the disease [9]. This approach is known as radiomics. The emergence of radiomics has broaden the scope of routine medical imaging in clinical oncology. Some previous studies have shown that biomarkers based on quantitative radiomics is associated with clinical prognosis across a range of cancer types [10–13].

To our best of knowledge, the potential role of radiomics signature as a prognostic biomarker for advanced NPC has not been explored. In the present study, we performed multivariate analyses to determine whether the radiomics signature is an independent predictor of the progression-free survival of NPC patients.

## RESULTS

### Patient and tumor characteristics

Patient and tumor characteristics in the discovery and validation sets are listed in Table 1. No differences were found between the training and validation cohorts in terms of age, gender, overall stage, histology, or follow-up time (p = 0.129-0.935). The median follow up time was 39.5 months (range, 3-89 months).

**Table 1:** Patient and tumor characteristics in the discovery and validation sets

	Discovery set (N = 70)	Validation cohort (N = 30)	p-value
Sex			
Male	50 (71.4%)	26 (86.7%)	0.129
Female	20 (28.6%)	4 (13.3%)	
Age (years)			
Median (IQR)	42 (36.5-51.00)	44 (36.0-51)	0.935
≤40	33 (47.0%)	13 (43.4%)	
40-50	19 (27.0%)	9 (30%)s	
>50	18 (26.0%)	8 (26.6%)	
Overall stage			
III	45 (64.3%)	22 (73.4%)	0.488
IV	25 (35.7%)	8 (26.6%)	
Histology			
WHO type I	0	0	0.361
WHO type II	3 (4.3%)	3 (10%)	
WHO type III	67 (95.7%)	27 (90%)	
Pretreatment hemoglobin (g/L)			
Median (IQR)	174 (142-234)	142 (134-153)	**<** 0.001
≤156	26 (37%)	24 (80%)	
>156	44 (63%)	6 (20%)	
Pretreatment platelet counts (10^9^/L)			
Median (IQR)	137(123-169)	234 (180-297)	< 0.001
≤158	49 (70%)	5 (17%)	
>158	21 (30%)	25 (83%)	
Follow-up time (mo)			
Median (IQR)	39.5 (24-58)	39.5 (29-50)	0.722

Data are n (%) unless otherwise indicated. *Histology was categorized according to the WHO Classification. IQR: inter-quartile range; type I: keratinizing; type II: non-keratinizing differentiated; type III: non-keratinizing undifferentiated.

### Construction of the rad-score based radiomics signature

A total of 970 radiomics features were extracted from MR images (485 features from T_2_-w images and the remaining 485 from CET_1_-w images). The five textural features with a non-zero coefficient in the lasso-Cox regression model were as follows: CET_1_-w_5_GLCM_correlation, T_2_-w_1_GLRLM_SRLGLE, CET_1_-w_6_GLCM_ IMC1, T_2-_w_1_GLCM_inverse_variance, and T_2_-w_3_GLCM_homogeneity 1. The radiomics signature was constructed, with a Rad-score calculated by using the following formula:

Rad-score = 2.495 * T_2_-w_3_ GLCM_homogeneity 1

 + 1.474 * CET1-w_6_ GLCM _ IMC1

 - 1.203 * CET_1_-w_5_GLCM _correlation

 - 0.809 * T_2-_w_1_GLCM_inverse _variance

 - 3.839 * T_2_-w_1_GLRLM_SRLGLE

 - 7.995

**Figure 1:**
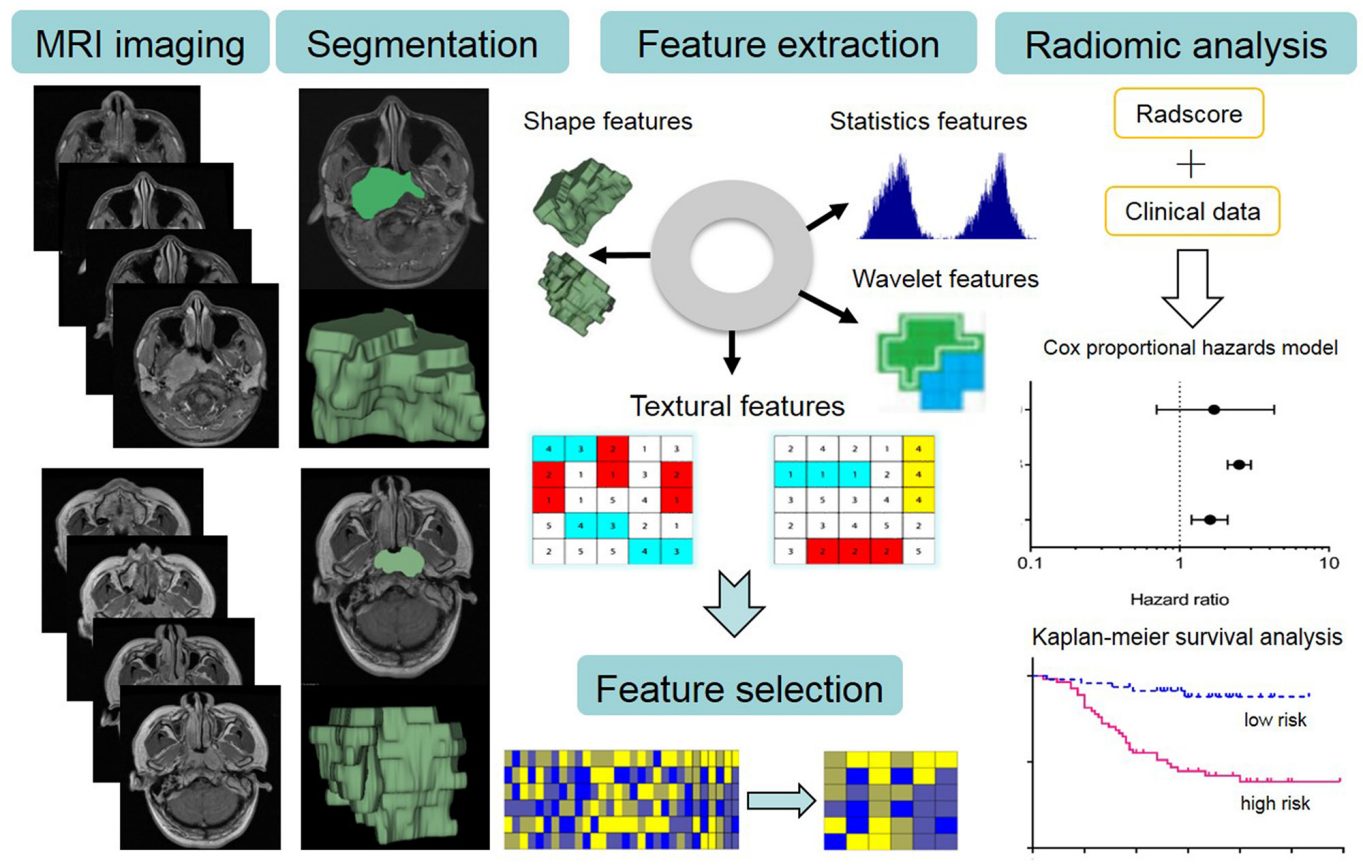
The image post-processing workflow Image segmentation is performed on contrast-enhanced T1-w and T2-w MRI images. Experienced radiologists contour the tumor areas on all MRI slices. Radiomics features are extracted from within the defined tumor contours on the MRI images, quantifying tumor intensity, shape, texture, and wavelet filter. Least absolute shrinkage and selection operator (lasso) regression was used to select features that were most associated with PFS. For the radiomic analysis, multivariate Cox proportional hazards models and stratified Kaplan-Meier analyses were performed to assess the prognostic value of radiomics signature.

The contribution of the selected parameters with their absolute value of regression coefficients is presented in the form of a histogram in Figure 1. We could observe that the absolute value of coefficient of feature T_2_-w_1_GLRLM_SRLGLE was the highest.

The optimum cutoff was the median of Rad-score. Accordingly, patients were divided into a high-risk group (Rad-score ≥ - 6.863) and a low-risk group (Rad-score <- 6.863).

### The correlation of radiomics features with tumor volume

The strength of the correlation coefficient was categorized as follows: 0-0.25 = little if any correlation, 0.26-0.49 = low correlation, 0.50-0.69 = moderate correlation, 0.70-0.89 = high correlation, and 0.90 to 1.0 = very high correlation. As a result, 69, 9, 18, 89, and 300 CET_1_WI-based features respectively show very high, high, moderate, low and little correlation with tumor volume. A total of 69, 9, 29, 111, and 267 T_2_WI-based features show very high, high, moderate, low and little correlation with tumor volume. The detailed information was provided in the Supplementary material.

### Multivariate analyses assessing the prognostic value of radiomics signature

Candidate prognostic factors including age, gender, overall stage, hemoglobin, platelet counts and radiomics signature were included in the multivariate Cox proportional hazards model. We developed model 1 (without radiomics signature) and model 2 (with radiomics signature) in the discovery set and then tested in the validation set. The results demonstrated that radiomics signature was a significant, independent predictor of PFS in the discovery set (HR = 5.14, 95% CI= 4.80-5.48, p < 0.001) and the validation set (HR = 7.28, 95% CI = 6.46-8.09, p = 0.015) (Table 2). However, the other clinical risk factors showed no significantly prognostic value in both model 1 and model 2 (all p-value for HR, > 0.05) (Table 2).

**Table 2:** Multivariate cox proportional hazards models in the discovery and validation sets

Variable	Model 1		Model 2	
	HR (95%CI)	p-value	HR (95%CI)	p-value
Sex				
discovery set	3.76 (3.01-4.50)	0.08	1.38 (0.58-2.18)	0.69
validation set	3.85 (3.07-4.63)	0.08	1.98 (1.37-2.9)	0.27
Age				
discovery set	0.99 (0.97-1.01)	0.52	1.01 (0.98-1.03)	0.80
validation set	0.99 (0.97-1.02)	0.82	0.99 (0.97-1.01)	0.62
Overall stage				
discovery set	1.35 (1.02-1.69)	0.36	1.04 (0.55-1.54)	0.93
validation set	0.80 (0.24-1.37)	0.70	1.62 (1.14-2.10)	0.31
Hemoglobin				
discovery set	1.00 (0.99-1.00)	0.34	0.99 (0.99-1.00)	0.19
validation set	1.01 (1.00-1.01)	0.39	0.99 (0.99-1.00)	0.25
Platelet counts				
discovery set	0.99 (0.99-1.00)	0.08	1.00 (0.99-1.00)	0.83
validation set	1.00 (0.99-1.00)	0.41	1.00 (0.99-1.00)	0.93
Rad-score				
discovery set	**---**	**---**	5.14 (4.80-5.48)	< 0.001
validation set	**---**	**---**	7.28 (6.46-8.09)	0.02

### Representative cases show heterogeneity is more important than tumor extent or T staging

A 39-year-old male patient, with stage of T2N2M0 and Rad-score of -6.599, who experienced disease progress at 3 months after treatment. Another 35-year-old male patient, with a more advanced stage of T3N2M0 but a smaller Rad-score of -7.156, no disease progress was observed after a follow-up of 39 months.

A 47-year-old male patient, with stage of T4N2M0 and Rad-score of -6.400, who experienced disease progress at 20 months after treatment. Another 43-year-old male patient, with a same stage of T4N2M0 but a smaller Rad-score of -7.157, no disease progress was observed after a follow-up of 60 months.

A 37-year-old male patient, with stage of T3N2M0 and Rad-score of -6.413, who experienced disease progress at 27 months after treatment. Another 40-year-old male patient, with same stage of T3N2M0 but a smaller Rad-score of -7.146, no disease progress was observed after a follow-up of 51 months.

### Stratified Kaplan-Meier analyses

**Figure 2:**
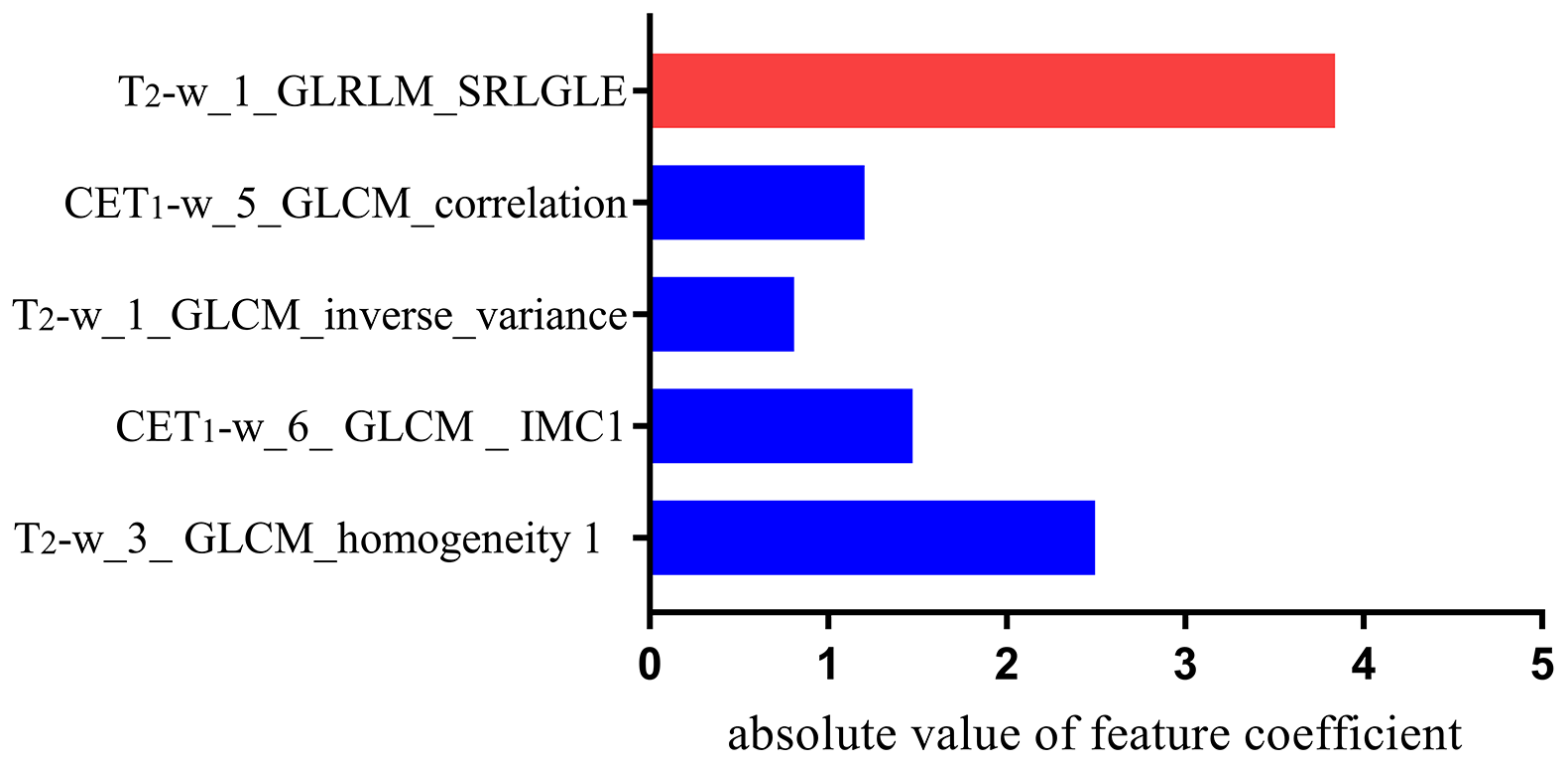
Histogram shows the role of individual textural features that contribute to the developed radiomics signature The features that contribute to the radiomics signature are plotted on the y-axis, with their absolute value of coefficients in the lasso Cox analysis plotted on the x-axis

**Figure 3:**
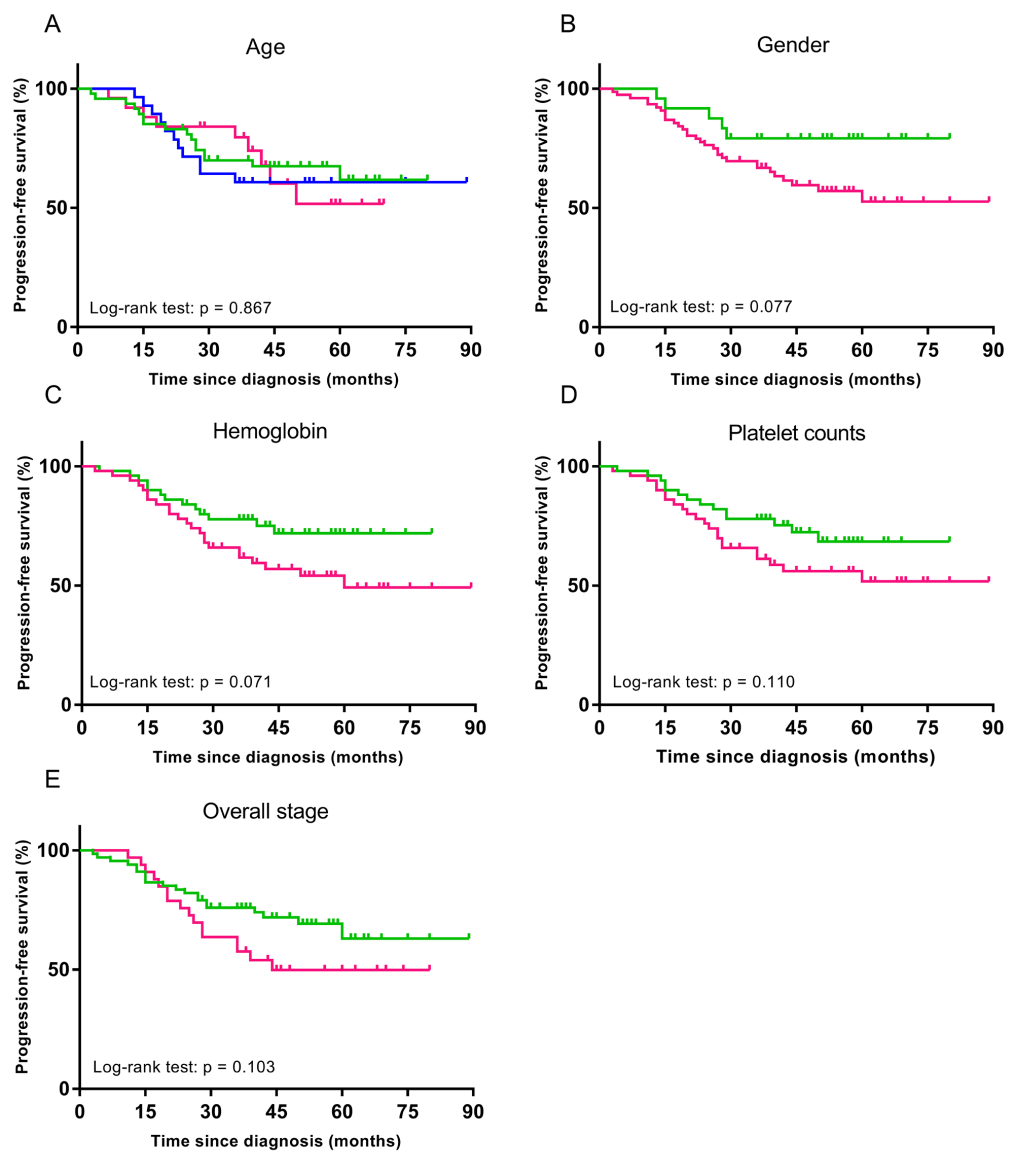
Stratified Kaplan-Meier analyses were performed to estimate progression-free survival in clinical subgroups **(A)** Green, blue and pink curves describe PFS of patients with age ≤ 40 years, 40-50 years, and > 50 years, respectively. **(B)** Green and pink curves of female and male patients, respectively. **(C)** Green and pink curves of patients with pretreatment hemoglobin ≤156 g/L and > 156 g/L, respectively. **(D)** Green and pink curves of patients with pretreatment platelet counts ≤ 158 × 109/L and > 158 × 109/L, respectively. **(E)** Green and pink curves of III and IV patients, respectively.

When patients were stratified by age (≤ 40 years, 40-50 years, or >50 years), gender (female or male), overall stage (III or IV), hemoglobin (≤ 156 g/L or >156 g/L), and platelet counts (≤ 158 × 10^9^/L or >158 ×10^9^/L), no differences were observed in patients’ PFS (p = 0.071-0.867) (Figure 2A-2E). However, when these patients were further stratified by Rad-score, differences in PFS were evident (for all, p < 0.003) (Figure 3A-3E). In particular, when patients were stratified by Rad-score alone, we could find that the PFS was significantly lower in low risk patients than high risk patients (log rank test, p < 0.0001) (Figure 3F).

## DISCUSSION

Radiomics applies advanced computational methods to convert medical images into a large number of quantitative descriptors of tumors [14]. In the present study, we used a high-throughput radiomic approach to automatically extract 970 quantitative MRI features and analyze their potential value for evaluating progression-free survival of 100 patients with advanced NPC. About 38% CET_1_WI-based and 45% T_2_WI-based features show low to very high correlation with tumor volume. Our results reveal that pre-treatment MRI-based radiomics signature is a nonivasive and cost-effective prognostic marker in advanced NPC patients. Our radiomic approach is based on comprehensive quantitative information derived from two different MRI sequences which comprise a multiparametric three-dimensional characterization of the entire tumor.

In radiomics the number of features greatly exceeds the number of patients and conventional regression techniques may produce unsatisfactory results [15]. Therefore, we used lasso, which was shown to be an effective machine-learning algorithm to avoid over-fitting and select features that most significantly associated with the outcome. The radiomics features obtained from lasso are generally accurate, and the regression coefficients of most features are shrunk towards zero during model fitting, making the model easier to interpret [16, 17]. In this present study, to develop the best radiomics signature, a total of 970 candidate features were reduced to a set of only five potential predictors by using a lasso logistic regression model. We selected five textural features as potential predictors, which were divided into two typical matrices: the Gray-level co-occurrence matrix (GLCM) and the Gray-level run-length texture matrix (GLRLM). GLCM is the matrix function that describes the distance and angle of each pixel. By calculating the correlation between two gray levels with certain directions and distances, GLCM can reflect integrated information about the direction, interval, amplitude, and frequency of images. GLRLM can quantify gray level runs in an image. A gray level run is defined as the length (number of consecutive pixels) that have the same gray-level value. In this study, texture analysis, consisting of a variety of mathematical techniques that can describe the grey-level patterns of an image, plays an important role in assessing the spatial organization of NPC tumors [18].

Our multivariate Cox proportional hazards model 1 and 2 suggested that radiomics signature was the only prognostic biomarker and other clinical data including age, gender, overall AJCC stage, pre-treatment hemoglobin, and platelet counts showed no prognostic value. Stratified Kaplan-Meier analyses demonstrated when the patients were stratified on the basis of clinical risk factors, no significant difference was observed in any subgroup. However, when these patients were further stratified by median Rad-score, shorter PFS was found in high-risk patients than in low-risk patients. Currently, the TNM staging system is used for risk stratification and treatment decision making. However, when patients were stratified by clinical disease stage, differences in PFS were evident within the individual stages, which suggests that heterogeneity was present in the survival outcomes. Thus, the TNM staging system may not be precise enough and its prognostic value was limited. As for the tumor staging, radiomic analysis has recently been demonstrated to be discriminative in esophageal cancer, colorectal cancer, and non-small cell lung cancer (NSCLC). In contrast to traditional clinical staging, which barely reflect the intra-tumor heterogeneity, our radiomic approach extracts textural features from the imaging characteristics of the entire tumor on medical images, thus provide a robust way to characterize the intra-tumor heterogeneity non-invasively. Currently, the intra-tumor heterogeneity has been reported to have pronounced effects on prognosis, and thus it is considered to be a potential prognosis factor [19]. This view fits our knowledge of cancer, in which malignant lesions consist of heterogeneous cell populations with distinct molecular and micro-environmental differences.

The clinical relevance of our study lies in the advancement of the noninvasive analysis and characterization of NPC, and in the extension of existing knowledge by novel putative imaging biomarkers that currently do not exist in clinical routine. Recent efforts focused on finding potential biomarkers for the prediction of survival of NPC patients, such as body mass index, hemoglobin, lactate dehydrogenase, neutrophil-to-lymphocyte ratio, and platelet counts, high sensitivity C-reactive protein [20, 21], but the clinical utility of these factors was limited and unclear. To date, the plasma cell-free EBV DNA titre remains the only biomarker with clinical utility in NPC [22]. However, the proportion of NPC patients whose tumors are associated with EBV DNA vary with geographic location, and there are a variety of assays for plasma EBV DNA [23].

Limitations of this study should be acknowledged. Firstly, we used a validation set that derived from the same institution as the discovery set, which prevented us from investigating the generalizability of the findings to other institutions and settings. Furthermore, all patients were in clinical stage III-IVb, which will limit the application of our method to low stage patients. Lastly, we did not consider interaction on an additive scale between two radiomic features on a certain outcome.. If interactions between individual texture features had been identified, the interaction terms that were most strongly associated with the outcome interactions would have been selected when we constructed the radiomics signature, and this could have improved prognostic performance.

Taken together, radiomic profiling provides a complementary perspective by unraveling previously hidden information from MRI, which is the imaging modality of choice in NPC and routinely performed throughout the disease, allowing noninvasive, comprehensive assessment of the complete three dimensional tumor volume and, by leveraging the results from the current study, emphasizes assessment of radiomic data for predicting survival outcomes of advanced NPC patients. Our MRI-based radiomics signature emerges as a putative imaging biomarker for the identification of patients who may at high risk for shorter PFS, advances the knowledge in the noninvasive characterization of NPC, and stresses the role of radiomics as a novel tool for improving decision support in cancer care at low cost [24].

## MATERIALS AND METHODS

### Patients

Retrospective data evaluation was approved by the local ethics committee and informed consent was waived. In all, 100 patients with diagnosed with NPC (III-IVb) were included in this study. All patients met the following criteria: (i). All patients were without evidence of recurrences at diagnosis. (ii). Patients underwent a pretreatment MRI scan. (iii). The minimum follow-up time to ascertain the progression-free survival (PFS) was 36 months. All patients were followed up every 1-3 months during the first 2 years, every 6 months in years 2-5, and annually thereafter. (iiii). All local recurrences were identified by flexible nasopharyngoscopy and biopsy and/or MRI imaging of the nasopharynx and skull base that showed progressive bone erosion and/or soft tissue swelling. Regional recurrences were confirmed by fine-needle aspiration or MRI scans of the neck. Distant metastases were diagnosed based on imaging methods including chest X-ray, whole-body bone scan, MRI, CT, PET/CT, and ultrasonography.

The identified patients were randomly allocated to a discovery and validation set (discovery set: n = 70; validation set: n = 30). Patient and tumor characteristics in the discovery set and validation set were compared in terms of age, gender, histology, pre-treatment hemoglobin, pre-treatment platelet counts, overall stage and follow-up time. Tumor staging was performed on the basis of the American Joint Committee on Cancer TNM Staging System Manual (7th Edition). The PFS was calculated from the diagnosis until tumor progression.

### MRI imaging

Images were acquired in the routine clinical workup using a 1.5 T MR system (Signa EXCITE HD, TwinSpeed, GE Healthcare, Milwaukee, WI, USA). The acquisition parameters were as follows: axial T_2_-weighted spin-echo images (TR/TE: 5000/85 msec, FOV = 23 × 23 cm, NEX = 2.0, Slice thickness = 4 mm, Spacing = 1.0 mm) and axial contrast- enhanced T_1_-weighted spin-echo images (TR/TE: 410/Min Full msec, FOV = 23 × 23 cm, NEX = 2.0, Slice thickness = 4 mm, Spacing = 1.0 mm).

### Image post-processing pipeline

Before image processing, we excluded images with artifacts, and then we performed image filtering: a process was applied to selectively extract features of diverse sizes and intensity variations. A Laplacian of Gaussian spatial band-pass filter was used, by turning the filter parameter between 1.0 and 1.5. The filter values of 0 indicated no filtration, 1.0 indicated degrees of fine texture, 1.5 and 2.0 indicated medium textures, while 2.5 indicated coarse texture. The Laplacian of Gaussian filter distribution is given by

G2(X,Y)=−1ππσ4(1−x2+y22σ2)e−(x2+y22σ2)

x, y denote the spatial coordinates of the pixel and σ is the value of filter parameter.

**Figure 4:**
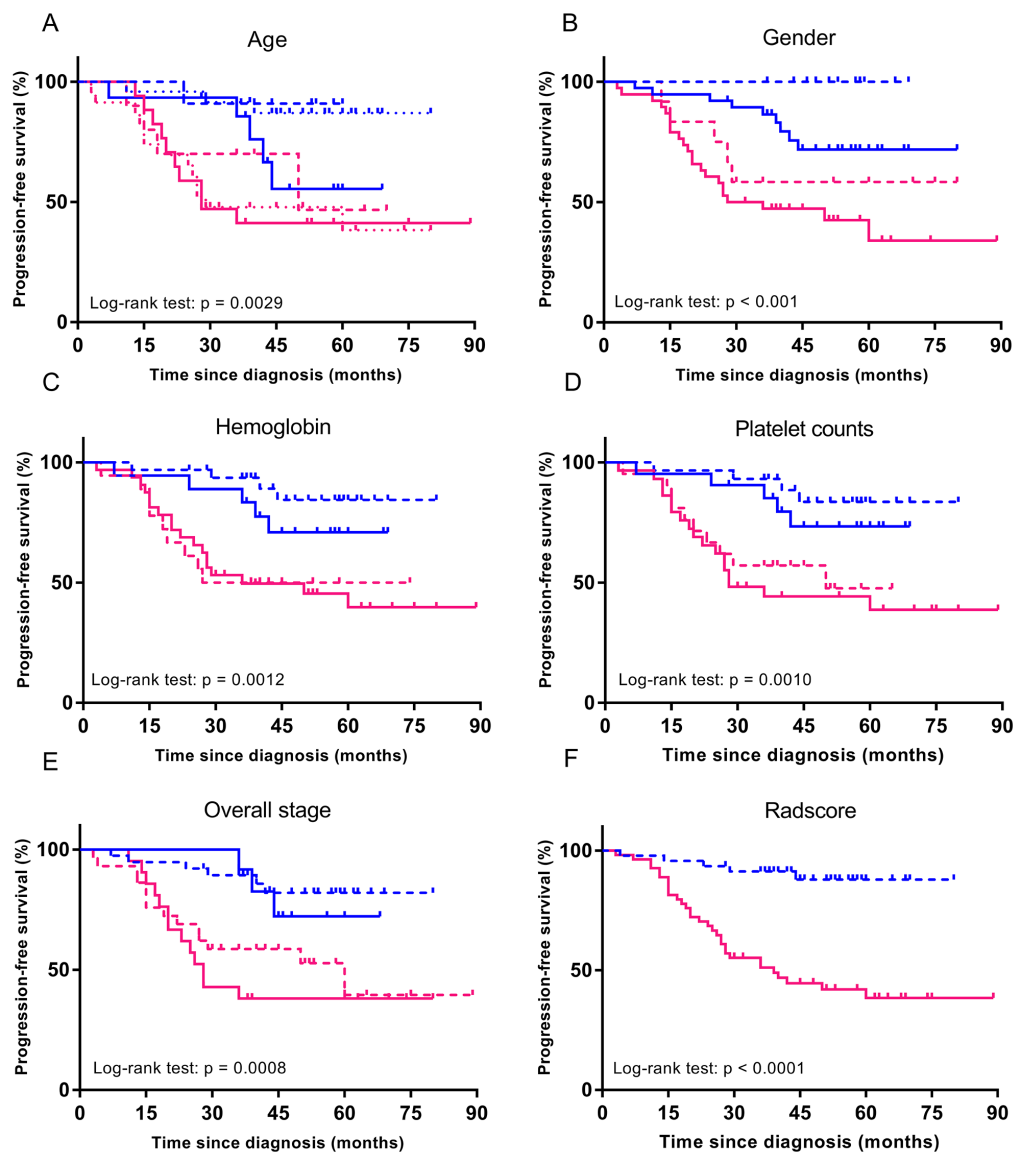
The image post-processing workflow Stratified Kaplan-Meier analyses were performed to estimate progression-free survival in various subgroups, **(A-F)** comparing high-risk patients and low-risk patients according to median Rad-score. Blue and pink curves describes PFS of low and high-risk patients.

Figure 4 depicts the image post-processing workflow. Axial T_2_-weighted (T_2_-w) and contrast-enhanced T_1_-weighted (CET_1_-w) Digital Imaging and Communications in Medicine (DICOM) images (512 by 512 pixels) for three-dimensional segmentation using algorithms implemented in an open source software ITK-SNAP (http://www.itk-snap.org). All manual segmentations of the tumor were performed by a radiologist who had 10-year of experience, and each segmentation was validated by a senior radiologist, who had 20-year of experience in NPC diagnosis. The region of interest (ROI) covered the whole tumor and was delineated on both the axial T_2_-w and CET_1_-w images on each slice (Supplementary Figure).

Quantitative features included first-order features, volume and shape features, textural features, and wavelet features. For each image volume, eight decompositions were calculated using discrete wavelet transformations which effectively decouple textural information by decomposing the original image in three directions (x, y, z). The size of each decomposition is equal to the original image and each decomposition is shift invariant. All feature extraction methods were implemented using Matlab 2014a (MathWorks, Natick, MA, USA). The least absolute shrinkage and selection operator (lasso) logistic regression was used to select the most strong radiomics features associated with the patients’ PFS. Radiomics signature were built using Rad-score. The Rad-score was calculated for each patient as a linear fitting of selected features that were weighted by their respective coefficients.

### Statistical analysis

Subsequent analysis was performed using R version 3.2.3 (R Foundation for Statistical Computing). All radiomic features (n = 970) were normalized by transforming the data into new scores with a mean of 0 and a standard deviation of 1 (z-score transformation). The package ‘glmnet’ was used for Lasso logistic regression model. The correlation of Radiomics features with tumor volume was calculated using spearman correlation analysis. A multivariate Cox proportional hazards model (backward step-down selection; the Akaike information criterion) were used to evaluate the performance of the clinical and radiomic predictors for stratifying PFS (separately assessed for both the discovery and validation set). Stratified Kaplan-Meier analyses were performed to explore the potential association of the radiomics signature with the PFS using subgroups within clinical-pathologic risk factors from the whole data set. The subgroups included age (≤ 40 years, 40-50 years, or >50 years), gender (female or male), overall stage (III or IV), hemoglobin (≤ 156 g/L or >156 g/L), and platelet counts (≤ 158 × 10^9^/L or >158 ×10^9^/L). The survival differences were compared using log-rank tests. The differences in age, gender, overall stage, histology, hemoglobin, platelet counts, and follow-up time between discovery set and validation set were assessed by using an independent samples t test, Chi-square test, or Mann-Whitney U test, where appropriate. All statistical tests were two-sided, and p-values of < 0.05 were considered significant.

## SUPPLEMENTARY MATERIALS FIGURE AND TABLE





## Abbreviations

NPC: nasopharyngeal carcinoma
PFS: progression-free survival
MRI: magnetic resonance imaging
PACS: picture archiving and communication system
AJCC: american joint committee on cancer
Lasso: least absolute shrinkage and selection operator
CI: confidence interval
HR: hazard ratio
FOV: field of view
NEX: number of excitations
TR: repetition time
TE: echo time
CET_1_WI: contrast-enhanced T_1_-weighted imaging
T_2_WI: T_2_-weighted imaging
GLCM: Gray-level co-occurrence matrix
GLRLM: Gray-level run-length texture matrix
