# Supplementary material for: Exploration and validation of radiomics signature as an independent prognostic biomarker in stage III-IVb nasopharyngeal carcinoma
Source: Oncotarget. 2017 Aug 24;8(43):74869–79. doi: 10.18632/oncotarget.20423 (PMC5650385; doi:10.18632/oncotarget.20423)
Supplement: Supplementary file 1 [file oncotarget-08-74869-s001.pdf]

Exploration and validation of radiomics signature as an independent prognostic biomarker in stage III-IVb nasopharyngeal carcinoma

SUPPLEMENTARY MATERIAL

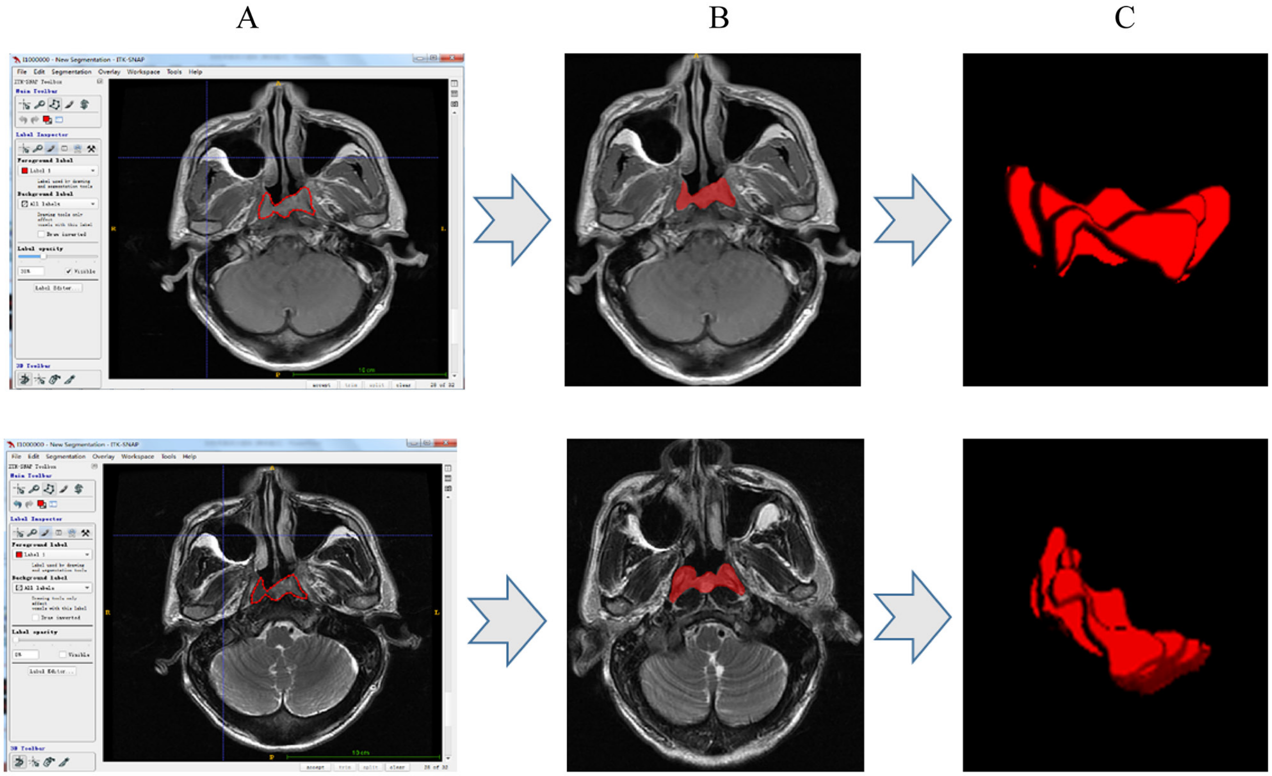

Supplementary Figure 1:Three-dimensional image segmentation. (A) The region of interest (ROI) was was delineated on both the axial T2-w and CET1-w images on each slice using ITK-SNAP; **(B)** Enter key was clicked to cover the whole tumor; **(C)** Three-dimensional images were obtained.

Supplementary Table 1: The association of Radiomics features with tumor volume.

See Supplementary File 1
